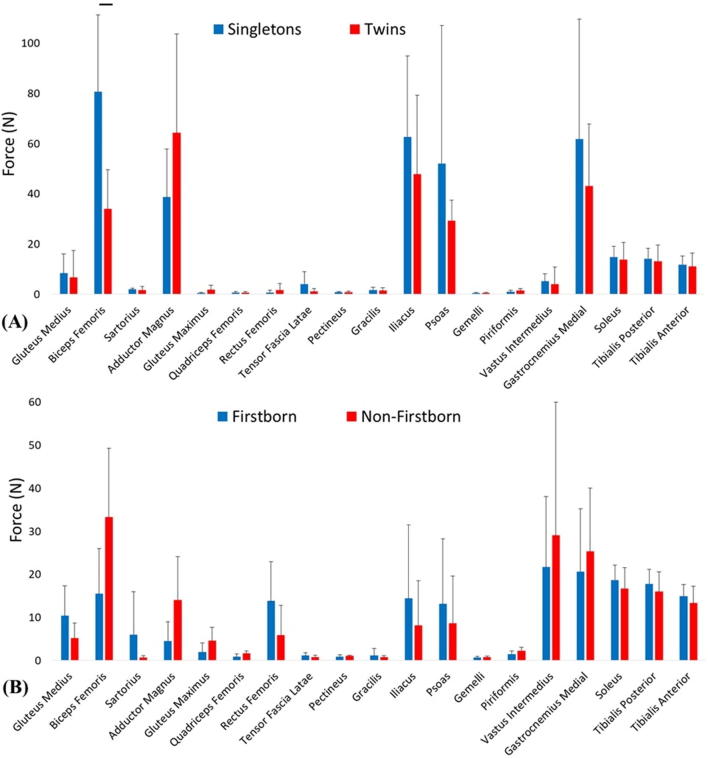# Altered biomechanical stimulation of the developing hip joint in presence of hip dysplasia risk factors

**DOI:** 10.1016/j.jbiomech.2018.07.016

**Published:** 2018-09-10

**Authors:** Stefaan W. Verbruggen, Bernhard Kainz, Susan C. Shelmerdine, Owen J. Arthurs, Joseph V. Hajnal, Mary A. Rutherford, Andrew T.M. Phillips, Niamh C. Nowlan

**Affiliations:** a Department of Bioengineering, Imperial College London, London, UK; b Department of Computing, Imperial College London, London, UK; c Department of Radiology, Great Ormond Street Hospital, London, UK; d UCL Great Ormond Street Institute of Child Health, London, UK; e Department of Biomedical Engineering & Centre for the Developing Brain, School of Biomedical Engineering and Imaging Science, Kings College London, London, UK; f Department of Perinatal Imaging and Health & Centre for the Developing Brain, School of Biomedical Engineering and Imaging Science, Kings College London, London, UK; g Department of Civil and Environmental Engineering, Imperial College London, London, UK

**Keywords:** Musculoskeletal development, Joint biomechanics, Cine MRI, Developmental dysplasia of the hip, Computational model, External cephalic version, ECV

## Abstract

Fetal kicking and movements generate biomechanical stimulation in the fetal skeleton, which is important for prenatal musculoskeletal development, particularly joint shape. Developmental dysplasia of the hip (DDH) is the most common joint shape abnormality at birth, with many risk factors for the condition being associated with restricted fetal movement. In this study, we investigate the biomechanics of fetal movements in such situations, namely fetal breech position, oligohydramnios and primiparity (firstborn pregnancy). We also investigate twin pregnancies, which are not at greater risk of DDH incidence, despite the more restricted intra-uterine environment. We track fetal movements for each of these situations using cine-MRI technology, quantify the kick and muscle forces, and characterise the resulting stress and strain in the hip joint, testing the hypothesis that altered biomechanical stimuli may explain the link between certain intra-uterine conditions and risk of DDH. Kick force, stress and strain were found to be significantly lower in cases of breech position and oligohydramnios. Similarly, firstborn fetuses were found to generate significantly lower kick forces than non-firstborns. Interestingly, no significant difference was observed in twins compared to singletons. This research represents the first evidence of a link between the biomechanics of fetal movements and the risk of DDH, potentially informing the development of future preventative measures and enhanced diagnosis. Our results emphasise the importance of ultrasound screening for breech position and oligohydramnios, particularly later in pregnancy, and suggest that earlier intervention to correct breech position through external cephalic version could reduce the risk of hip dysplasia.

## 1 Introduction

Fetal movements during pregnancy are a natural part of the development process, and are detectable from 10 gestational weeks using ultrasound (de Vries and Fong, 2006). Sudden changes in fetal movements are known to be a strong predictor of fetal health, particularly approaching term, where decreases in fetal movements have been linked to poor fetal outcomes, such as low birth weight or preterm delivery (Dutton et al., 2012, O'Sullivan et al., 2009), and even stillbirth (Efkarpidis et al., 2004, Whitworth et al., 2011). Fetal movements are also known to play a significant role in normal development of the musculoskeletal system (reviewed in Nowlan, 2015). In cases of neuromuscular disorders with severely reduced or absent fetal movement, patients present with skeletal malformations such as joint fusions, craniofacial abnormalities and hypo-mineralised bones (Aronsson et al., 1994, Rodríguez et al., 1988a, Rodríguez et al., 1988b). Clinical evidence for the importance of fetal movements for skeletal development has been reinforced by studies of animal models, with abnormal joint conditions arising in both immobilised chick embryos and mutant mouse embryos with reduced or absent muscle activity (Kahn et al., 2009, Nowlan et al., 2010a, Nowlan et al., 2010b, Nowlan et al., 2014, Roddy et al., 2011). Indeed, a recent bioreactor study demonstrated that there is a dose-dependent relationship between movement and joint shape development in the chick embryo (Chandaria et al., 2016). Furthermore, a study of muscle-less mouse embryos observed down-regulation of key developmental regulatory genes in fetal skeletal rudiments when muscle forces were absent (Rolfe et al., 2014). Taken together, this evidence demonstrates that mechanical forces generated by fetal movements are required for normal prenatal musculoskeletal development, especially in the case of joint shape.

A relatively common developmental joint abnormality, observed in approximately 1.3 per 1000 live births, is known as developmental dysplasia of the hip (DDH) (Leck, 2000), and is indicated by instability, malformation or dislocation of the joint formed by the femoral head and the acetabulum of the pelvis (Weinstein, 1987). DDH has major implications for patient health, necessitating use of a harness postnatally, or possibly even surgery, to correct the shape. There are additional long term implications of the condition, as joint shape is strongly linked to risk of osteoarthritis in later life (Sandell, 2012). Despite known genetic risk factors for DDH, such as female gender and positive family history (Homer and Hickson, 2000), the other major risk factors relate to a more restrictive intra-uterine environment for fetal movements. The primary environmental risk factors are fetal breech position (Muller and Seddon, 1953), low amniotic fluid volume (oligohydramnios) (Hinderaker et al., 1994), and neuromuscular disorders (Homer and Hickson, 2000). Furthermore, breech position was recently linked to lower bone mineral content in neonates, persisting in hip up to 4 years of age (Ireland et al., 2018). While not an abnormal intra-uterine condition, primiparous (firstborn) pregnancies also carry a significantly greater risk of DDH when compared to subsequent pregnancies (Chan et al., 1997, de Hundt et al., 2012, Stein-Zamir et al., 2008, Yiv et al., 1997), which may be related to greater uterine muscle tone in primiparity (Wilkinson, 1963). Interestingly, despite less available uterine space in twin pregnancies, the incidence of DDH in twins is no higher than in singletons (De Pellegrin and Moharamzadeh, 2010, Dezateux and Rosendahl, 2007). However, little is known of how the biomechanics of fetal movements change with intra-uterine environment or fetal position.

Recent advancements in MRI technology provide a novel method through which movements of an entire fetus can be directly observed, known as cine-MRI scans (Guo et al., 2006, Hayat et al., 2011). By tracking normal fetal movements from this type of scan and by applying a series of computational techniques, including finite element (FE) analysis and musculoskeletal modelling, we previously quantified fetal kick force and associated intramuscular forces for the first time (Verbruggen et al., 2016), and characterised the changes in biomechanical stress and strain in the fetal skeleton over gestation (Verbruggen et al., 2018). In this study, we investigate biomechanical stimuli in the developing hip joint for intra-uterine situations that increase the risk of DDH, as well as for twins, which counterintuitively don’t have an increased risk of the condition. We hypothesise that fetal kicking, and the resulting stress and strain in the fetal skeleton, are altered in conditions associated with increased risk of DDH when compared to normal intrauterine conditions.

## 2 Materials and methods

### 2.1 Data acquisition

**Fig. 1:**
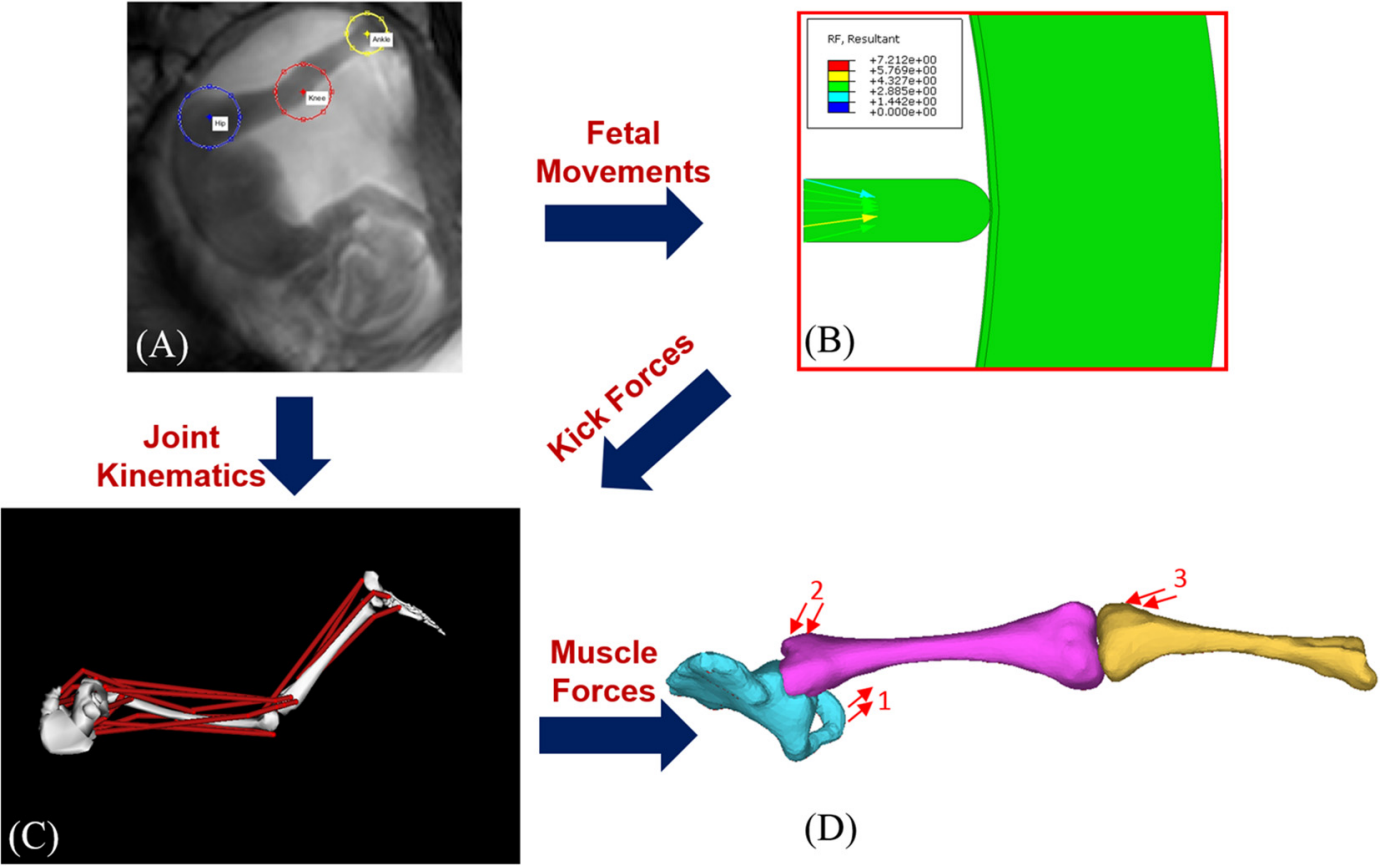
Flowchart outlining the computational pipeline used for this study. Computational methods applied comprise: (A) tracking of fetal joint kinematics, (B) finite element modelling of reaction force resulting from fetal movements against the uterine wall, (C) musculoskeletal modelling to predict muscle forces, (D) application of muscle forces to finite element models of fetal geometries (forces for adductor magnus (1), gluteus maximus (2), and biceps femoris (3) shown here for illustratory purposes). Adapted from Verbruggen et al. (2018).

**Fig. 2:**
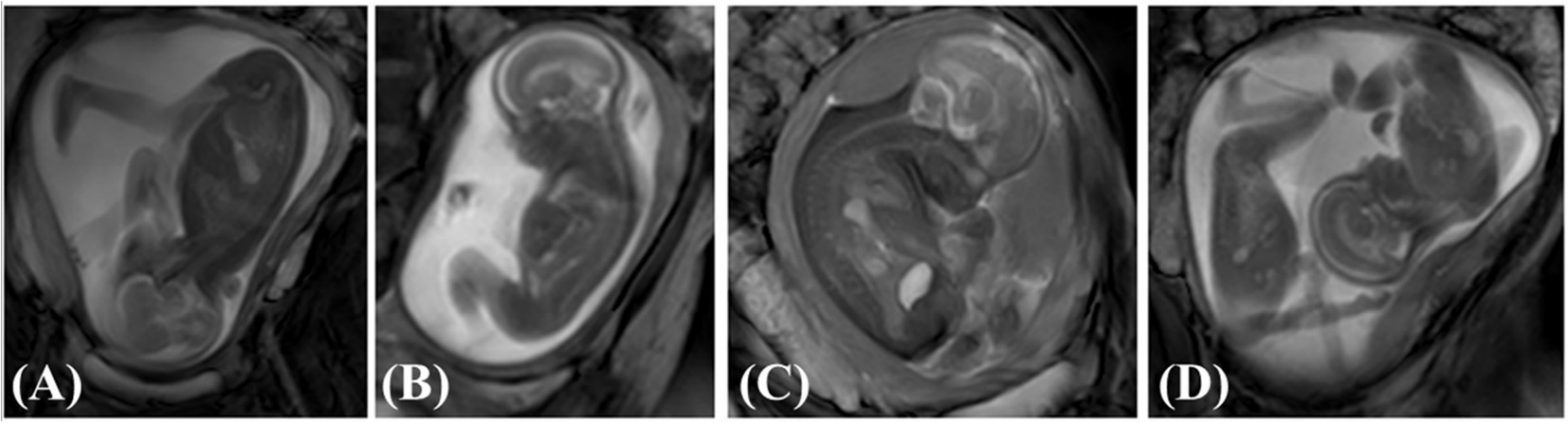
Stills from cine-MRI scans of (A) normal singleton, (B) breech position, (C) oligohydramnios and (D) twin pregnancies.

A database of fetal cine-MRI scans acquired from archived data of previous pregnancies at Hammersmith Hospital and St. Thomas’ Hospital (both London, UK) was retrospectively analysed for those which included clear in-plane extension-flexion fetal kicks in a range of conditions (Fig. 1). A total of 341 scans from different individuals were examined, of which the following were chosen at 20 weeks gestational age: breech position (*n* = 5) and twin pregnancies (*n* = 5). Two groups of healthy, cephalic singletons (*n* = 5 each) were also analysed at 20 and 30 weeks, with these groups reported in our previous study (Verbruggen et al., 2018). Separately, a group of nine oligohydramnios scans at approximately 30 weeks were obtained, with kick movements observed in three scans (*n* = 3). Scans from each of these conditions can are shown in Fig. 2. Finally, a group of first-born fetuses (*n* = 6) was selected from the larger cohort of 341 scans and compared to a group of second, third or fourth born fetuses (*n* = 6), all at approximately 20 weeks gestational age. All women had given prior consent for scans to be used in research as part of larger ethically approved trials (Hammersmith Hospital Research Ethics Committee/MHRA approval for IEH award 102431).

Separately, post mortem MR scans of fetal leg bones at approximately 20 and 30 weeks gestational age were obtained from the radiology information system (RIS) at Great Ormond Street Hospital (London, UK), as detailed previously (Verbruggen et al., 2018). The sample sizes and controls used for each intra-uterine situation under investigation are described in Table 1.

**Table 1:** Description of the risk factors for which kicks were modelled, as well as the intra-uterine conditions as controls use for each case.

Risk Factor (n)	Test Condition (n)	Gestational Age
Breech position (5)	Cephalic position (5)	20 Weeks
Twins (5)	Singletons (5)	20 Weeks
Oligohydramnios (3)	Healthy (5)	30 Weeks
Firstborn (6)	Non-firstborn (6)	20 Weeks

### 2.2 Computational methods

Stress and strain in the fetal skeleton were quantified using a previously developed computational pipeline, as shown in Fig. 1. Firstly, the movements of individual joints *in utero* were tracked from cine-MR scans. Uterine dimensions and uterine wall deflection due to fetal kicking were also measured. Secondly, the reaction force generated by a fetal kick against the uterine wall was calculated using an FE model of the intra-uterine mechanical environment (Verbruggen et al., 2017). Thirdly, these results were applied as inputs for a scaled musculoskeletal model of the fetal lower limb developed in OpenSim (Version 2.4) (Delp et al., 2007, Verbruggen et al., 2016, Verbruggen et al., 2018), with muscle forces outputted from OpenSim alongside their lines of action (van Arkel et al., 2013). Finally, the biomechanical stimulation (i.e. stress and strain) of the fetal lower limb during kicking *in utero* was predicted using a pair of FE models of the fetal leg bones at 20 and 30 weeks gestational age, generated from post-mortem MR scans, as described previously (Verbruggen et al., 2018). Stress and strain values within the hip joint (proximal femur and acetabulum) were recorded as the 95th percentile values.

### 2.3 Statistical analysis

Depending on uterine condition and availability of data, three (oligohydramnios), five (breech, healthy cephalic) or six (firstborn, non-firstborn) cine-MR scans were analysed. The predicted muscle forces for each cine sequence were then applied to two fetal skeletal geometries, resulting (*n* = 6, 10 or 12 data points per group). All data are expressed as mean ± standard deviation. Normality was checked and statistical differences between groups were determined using an ANOVA analysis and a Tukey’s post-hoc test, with statistical significance defined as *p* < 0.05 (SPSS, IBM, New York, U.S.).

## 3 Results

The findings of the current study are described by intra-uterine situation in this section. The average fetal femur and tibia lengths, uterine dimensions and kick durations for each scenario are given in Table 2.

**Table 2:** Measurements and results arranged by intra-uterine condition, including: femur and tibia length, uterine major and minor axes, uterine wall displacement, kick reaction force, observed knee joint angles at end of kick, and change in joint angle during kick.

Group	Femur Length (mm)	Tibia Length (mm)	Uterine Major Axis (mm)	Uterine Minor Axis (mm)	Uterine Wall Displacement (mm)	Kick Reaction Force (N)	Extended Knee Joint Angle (°)	Change in Joint Angle during Extension (°)
Cephalic singletons, 20 Weeks	58.45 ± 9.11	56.14 ± 4.22	217.19 ± 42.74	163.03 ± 17.12	11.78 ± 4.72	38.85 ± 13.08	31.54 ± 8.24	23.62 ± 10.34
Twins	38.03 ± 2.49	35.72 ± 4.31	240.66 ± 34.07	161.75 ± 19.44	12.68 ± 0.81	47.13 ± 4.62	18.52 ± 26.77	20.14 ± 19.04
Breech	40.35 ± 4.11	38.59 ± 4.50	163.14 ± 17.15	88.93 ± 23.44	6.06 ± 0.69	22.68 ± 2.76	22.29 ± 21.71	18.81 ± 10.13
Cephalic singletons, 30 Weeks	61.37 ± 16.03	55.92 ± 9.31	236.29 ± 21.16	178.29 ± 23.36	11.52 ± 1.47	46.64 ± 5.30	21.89 ± 6.87	27.24 ± 6.79
Oligohydramnios	45.01 ± 2.85	39.33 ± 2.72	157.98 ± 13.57	79.48 ± 8.61	3.53 ± 0.15	5.50 ± 0.25	11.72 ± 3.65	1.99 ± 0.15
Firstborn	37.92 ± 4.51	37.31 ± 4.02	146.91 ± 9.14	99.25 ± 15.73	7.11 ± 1.59	27.40 ± 5.88	17.87 ± 13.32	15.47 ± 10.47
Non-firstborn	37.56 ± 4.18	37.75 ± 1.96	156.66 ± 6.16	81.39 ± 32.56	10.62 ± 1.17	40.08 ± 4.51	33.86 ± 18.85	9.76 ± 7.41

### 3.1 Fetal breech position

**Fig. 3:**
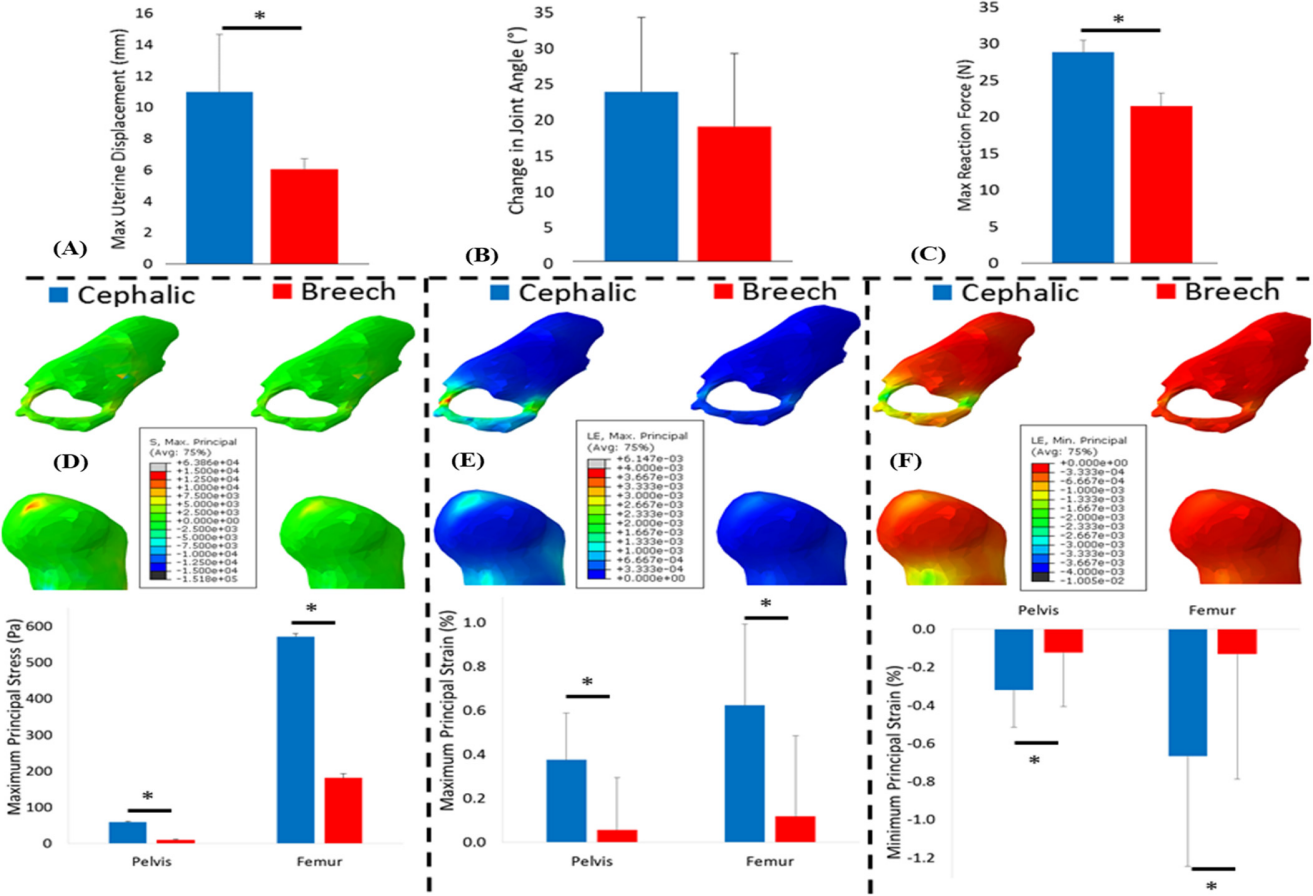
Kick forces, stress and strain resulting from fetal movements in cephalic and breech pregnancies. Average results for cephalic and breech pregnancies, for: (A) uterine wall displacement, (B) change in joint angle, (C) uterine reaction force, (D) maximum principal stress, (E) maximum principal strain and (F) minimum principal strain. The mean and standard deviation of each group are plotted. ^*^ Indicates statistical significance (p ≤ 0.05).

**Supplementary Fig. 1:**
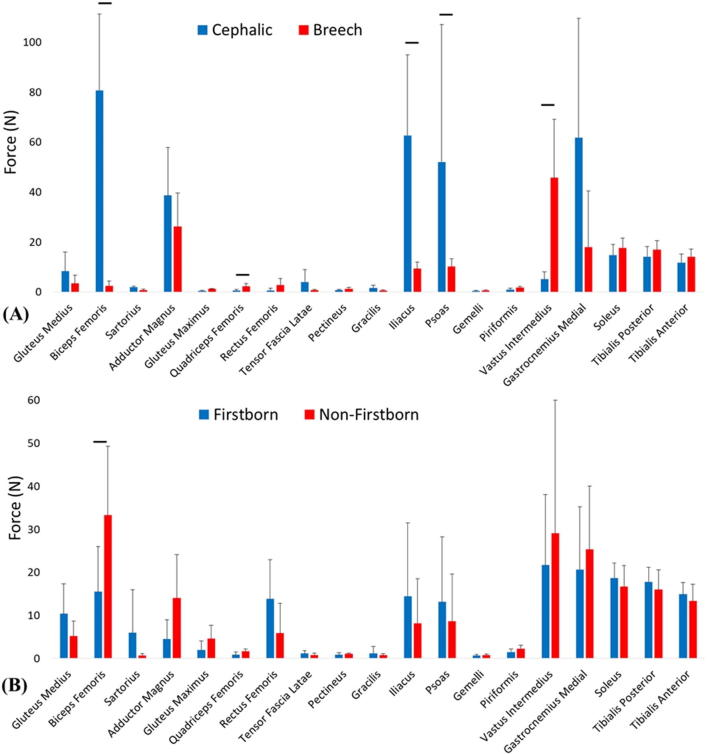


The average displacement of the uterine wall due to the observed kicks was found to be significantly lower in cases of breech presentation when compared to cephalic presentation, resulting in significantly lower kick force for breech position (Fig. 3 and Table 2). The average change in joint angle during fetal extension kick was lower in breech cases, though not significantly (Fig. 3 and Table 2). Large variations were observed in predicted muscle forces, though notably lower forces were observed in some muscle types in breech cases (see Supplementary Fig. 1). In both cephalic and breech cases, concentrations of maximum principal stress and strain were observed on the hip joint surface where contact between fetal bones occurred (see Fig. 3). Maximum principal stress was found to decrease significantly (by approximately two thirds) in the hip joint for breech cases, when compared to cephalic, with stress noticeably lower in all regions of the pelvis and femur (Fig. 3). Similarly, maximum and minimum principal strains were found to decrease significantly in magnitude for breech when compared to cephalic cases.

### 3.2 Oligohydramnios

**Fig. 4:**
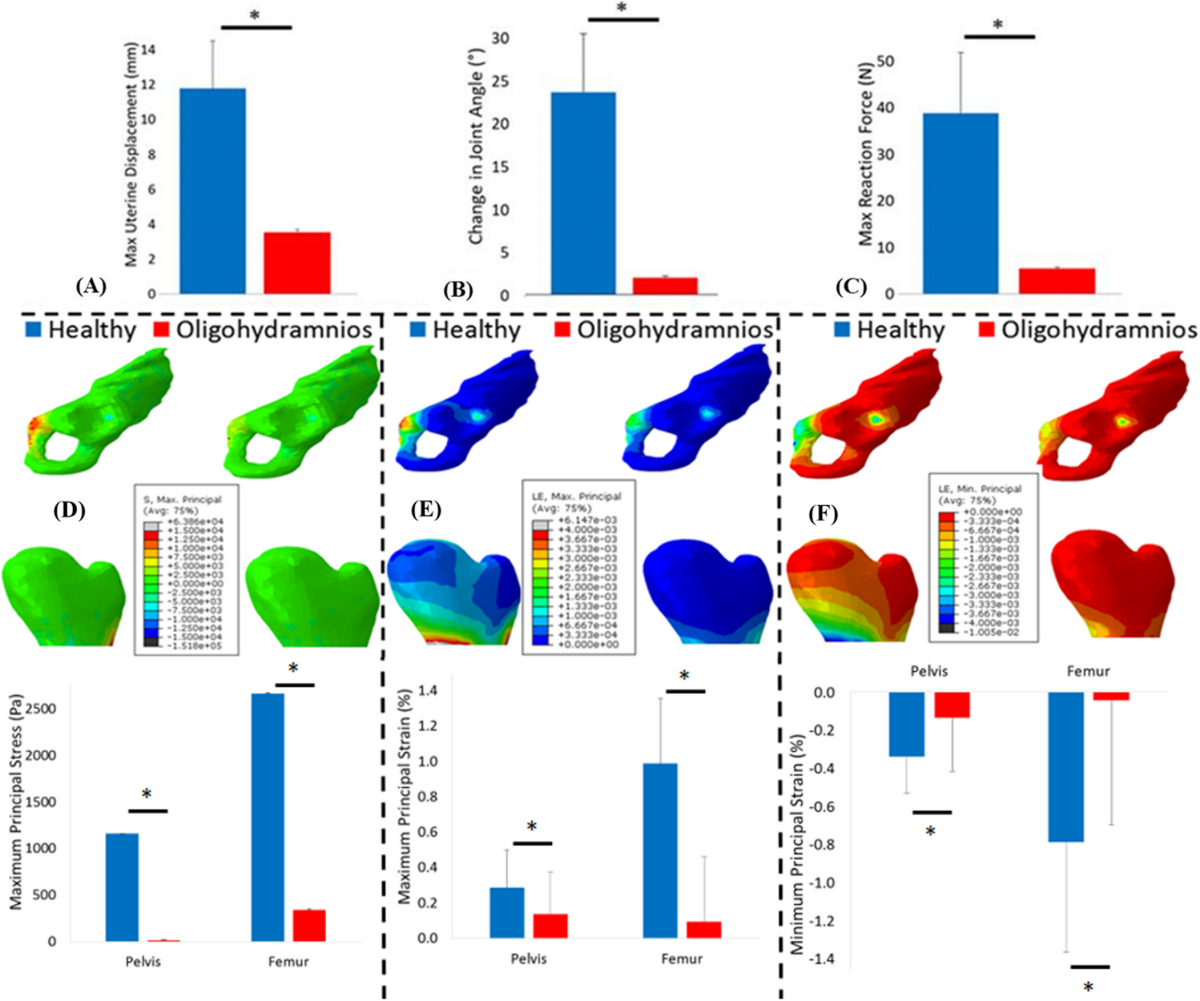
Kick forces, stress and strain resulting from fetal movements in healthy and oligohydramnios pregnancies. Average results for healthy and oligohydramnios pregnancies, for: (A) uterine wall displacement, (B) change in joint angle, (C) uterine reaction force, (D) maximum principal stress, (E) maximum principal strain and (F) minimum principal strain. The mean and standard deviation of each group are plotted. ^*^ Indicates statistical significance (p ≤ 0.05).

The average displacement of the uterine wall was significantly lower in cases of oligohydramnios when compared to normal cephalic kicks, with corresponding significantly lower kick forces for oligohydramnios conditions (Fig. 4 and Table 2). Additionally, a significant decrease in change of joint angle during kicks was observed in oligohydramnios cases as compared to normal cases (Table 2). While large variations in muscle forces were observed, significantly lower forces in some muscles were predicted in oligohydramnios cases (see Supplementary Fig. 1). While distributions of stress and strain were similar in both normal and oligohydramnios cases (Fig. 4), significant and pronounced decreases in maximum principal stress, and maximum and minimum principal strain were observed in oligohydramnios when compared to normal cases.

### 3.3 Twin vs. singleton pregnancies

**Fig. 5:**
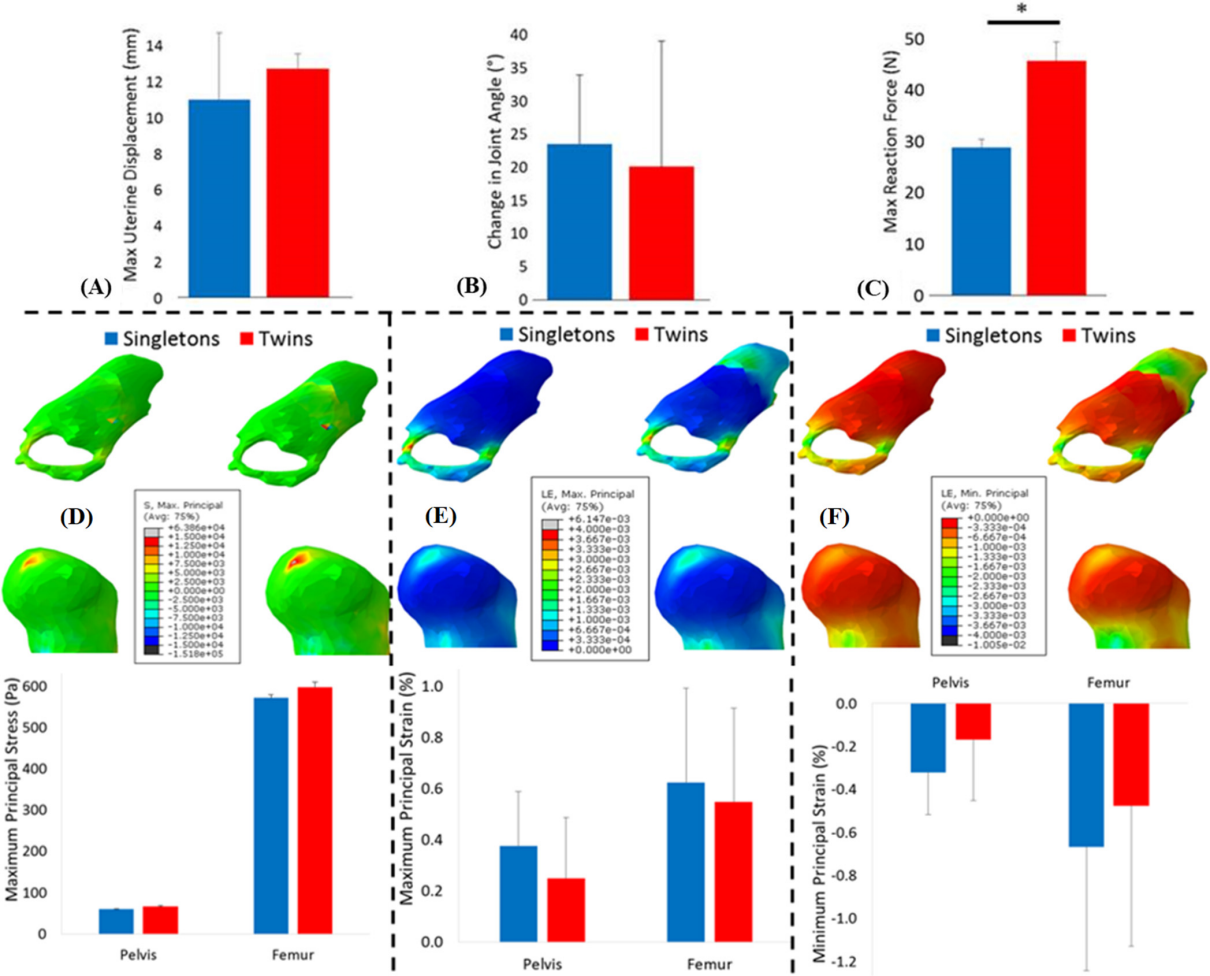
Kick forces, stress and strain resulting from fetal movements in singleton and twin pregnancies. Average results for singleton and twin pregnancies, for: (A) uterine wall displacement, (B) change in joint angle, (C) uterine reaction force, (D) maximum principal stress, (E) maximum principal strain and (F) minimum principal strain. The mean and standard deviation of each group are plotted. ^*^ Indicates statistical significance (p ≤ 0.05).

The average displacement of the uterine wall was not significantly different between twin and normal singleton kicks, while the average resulting kick force was higher in twins, though not significantly (Fig. 5 and Table 2). No significant differences were observed in joint angle change during kicks or in predicted muscle forces between twin and singleton pregnancies. Furthermore, no significant difference was found in either stress or strain in the hip joint between normal singleton and twin pregnancies.

### 3.4 Firstborn vs. non-firstborn pregnancies

**Fig. 6:**
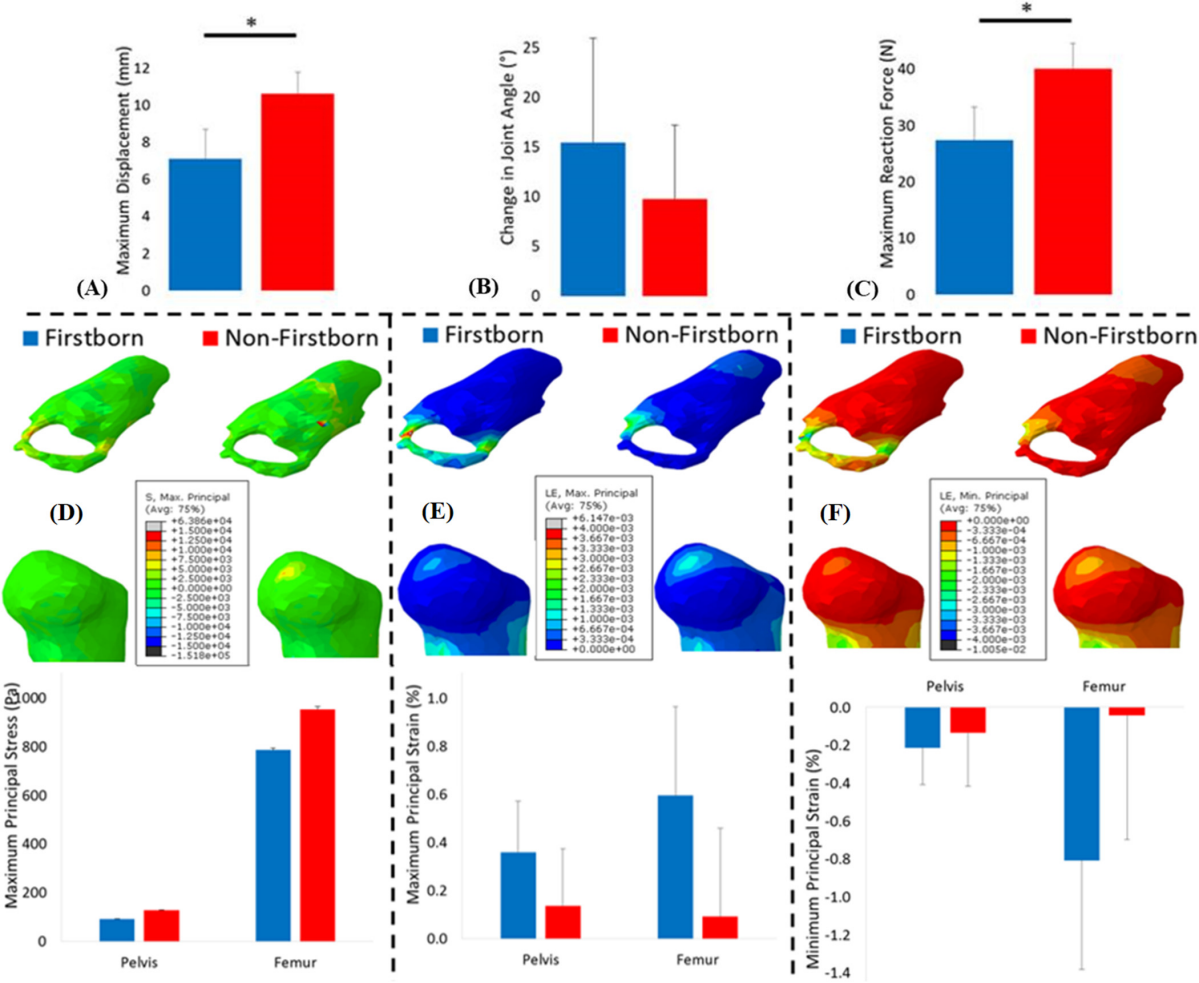
Kick forces, stress and strain resulting from fetal movements in firstborn and non-firstborn pregnancies. Average results for firstborn and non-firstborn pregnancies, for: (A) uterine wall displacement, (B) change in joint angle, (C) uterine reaction force, (D) maximum principal stress, (E) maximum principal strain and (F) minimum principal strain. The mean and standard deviation of each group are plotted. ^*^ Indicates statistical significance (p ≤ 0.05).

A small, but significant, decrease was observed in firstborn kick displacement when compared to non-firstborn (Fig. 6 and Table 2), and this resulted in significantly lower kick forces. No difference was observed in change of joint angle during kicking (Table 2), or in predicted muscle forces (see Supplementary Fig. 2). While no significant differences in either stress or strain were found when comparing firstborn with non-firstborn fetuses, a consistent trend of decreases in both stress and strain in firstborn fetuses was evident (Fig. 6).

## 4 Discussion

This study presents the first quantification of stress and strain as biomechanical stimuli in the fetal hip joint for a range of fetal positions and intra-uterine conditions, and is the first to establish a relationship between altered stress and strain magnitudes and known risk factors for DDH. In cases of fetal breech position and oligohydramnios, fetal kick force and stress and strain stimulation were significantly lower than in healthy cephalic conditions. Both of these conditions represent more restricted mechanical environments, with reduced range of movement and space for fetal leg movements, and are linked to an increased risk of DDH (Hinderaker et al., 1994, Muller and Seddon, 1953). By quantifying these movements and the resulting stress and strain for the first time, our results suggest that mechanobiology may explain the link between these conditions and risk of DDH. Similarly, firstborn fetuses are known to have a higher risk of DDH, and were found to generate significantly less displacement and kick force in our models. This may strengthen the theory that DDH is more likely when firstborn fetal movements are restricted by stiffer, less pliant maternal tissues, which have not before been stretched to the full extent during pregnancy (Nowlan, 2015, Wilkinson, 1963). Finally, it is known that twins are not at increased risk of DDH as compared to singletons, despite having reduced intra-uterine space available for movements (De Pellegrin and Moharamzadeh, 2010). Our measurements indicate that while twin fetuses are approximately 35% smaller than singletons at 20 weeks (using femoral length as an indicator of size), the combined mass of both fetuses would in fact be 30% larger than a singleton. We found no significant difference in kick force or stress and strain between twins and singletons, and furthermore, we observed similar kick displacement and reaction force in twin kicks, despite the limbs of twins being significantly smaller than singleton limbs. This may indicate additional or compensatory movements or stimulation in twins, and it is also possible that each twin would be exposed to a second source of biomechanical stimulation through the movements of, and interaction with, its companion.

As the pregnant human uterus is an experimentally inaccessible closed mechanical environment, a number of assumptions were required in order to conduct this research. A key limitation of this study is that these kicks were observed at a single time point of 20 or 30 weeks gestational age, due to availability of the cine-MRI data which is not routinely collected approaching full term. Therefore, the breech fetuses are less restricted than closer to term, when the risk of breech position for DDH is greater. However, our findings demonstrate that even at this early stage, breech position results in altered fetal biomechanics and hip joint stimulation, and we believe that the differences would be even more pronounced at later gestational ages. Additionally, a longitudinal study with multiple follow-up scans, such has recently been carried out for breech position in neonates, would shed new light on the link between breech and hip dysplasia (Ireland et al., 2018). In the case of oligohydramnios, these fetuses may be growth restricted, which could affect their ability to kick and play a role in the biomechanics characterised here. The uterine pressures used were measured in singleton pregnancies, and may therefore differ in twin pregnancies. However, the study from which the data was obtained involved invasive methods which would not be considered ethical in the present day, and therefore there is no feasible way to collect pressure data specific to twin pregnancies. From the large number of scans studied, only a small fraction demonstrated a clear, in-plane extension kick which could be tracked, resulting in relatively low sample numbers. Therefore, a larger dataset may provide a more representative sample of fetal biomechanics *in utero*. The Young’s modulus used for the uterine muscle tissue was obtained from studies of tissue excised during hysterectomy (Pearsall and Roberts, 1978), which could exhibit different behaviour to tissue *in vivo*, and in primiparity and oligohydramnios conditions. A sensitivity analysis of the material properties in the fetal skeleton model found that, while increasing or decreasing the elastic moduli of the mineralised and un-mineralised cartilage by 25% affected the magnitudes of stress and strain predicted, there was no effect on the trends of stress and strain for the various risk factors, which remained significantly lower for breech and oligohydramnios groups. Lastly, some unavoidable errors may have been introduced when applying muscle forces from the OpenSim model to the fetal geometries as not all anatomical landmarks for muscle attachment sites are fully developed (Geraldes et al., 2016).

The stress and strain characterised in this study illuminate a crucial missing link in our current understanding of the aetiology of DDH. The risk factors investigated in this study (i.e. breech position, oligohydramnios, primiparity) were previously identified based on correlation studies, without previous evidence of causation for these risk factors. Therefore, our findings provide the first scientific basis to explain these risk factors, observing altered fetal biomechanics in these conditions. Our results suggest that lower levels of stress and strain in the hip joint lead to an increased risk of hip dysplasia, indicating that changes in biomechanical stimuli affect joint development. Joint morphogenesis is ultimately a cell-driven process, with mechanically-mediated aspects of shape developing as fetal cartilage tissue responds to biomechanical stimuli, such as stress and strain. Given that this stimulation is impossible to investigate *in utero* experimentally in humans, our findings provide physiologically relevant stimuli for the first time, supplying new inputs for previously developed adaptive mechanobiological models of hip joint development and DDH (Giorgi et al., 2014, Giorgi et al., 2015).

These findings have clinical implications for diagnosis, prevention and intervention in cases where risk of DDH is increased. While early diagnosis is crucial for successful treatment (Sewell and Eastwood, 2011), DDH cannot currently be diagnosed prenatally using routine ultrasound scans. Similarly, the risk factors of late breech position and oligohydramnios are not specifically screened for using ultrasound in the UK National Health Service (NHS), with the second and final routine scan taking place at approximately 20 weeks. Our results emphasise the importance of targeted ultrasound screening specifically for breech position and oligohydramnios. Indeed, some units in the NHS now offer a 32 week growth scan, which could improve ultrasound screening of such risk factors. Interestingly, there may be the possibility of intervention to reduce incidence of DDH in breech cases. Fetal breech position has been found to be a greater risk for DDH from 37 weeks onwards when compared to before 37 weeks (Yiv et al., 1997). However, separate time points before 37 weeks have not been examined and it may be that breech position has negative effects even earlier (Yiv et al., 1997). Given the complications associated with a breech birth, current NHS policy recommends an external cephalic version (ECV), in which the fetus is manipulated out of breech position using manual external pressure, at 37 weeks. While current policy guidelines state that there is little benefit to performing ECV before 37 weeks, prior to the 1970s ECV was routinely performed earlier than 34 weeks (Hofmeyr and Kulier, 1996). It appears that this practice then changed to late term, with increasing evidence that ECV could be performed safely at term with the use of tocolysis (medical relaxation of the uterus) and the fact that the fetus can be delivered more readily in the event of complications (Hofmeyr and Kulier, 1996). However, a meta-analysis of the literature suggested that ECV intervention at 34–35 weeks, even though the fetus has space to return to breech position, may reduce breech presentation more successfully than ECV at 37 weeks (Hutton and Hofmeyr, 2006). Taken together with our findings, this suggests that targeted ultrasound scans at around 30 weeks and earlier ECV interventions could reduce the risk of DDH while not increasing the risks associated with ECV.

In summary, this research has characterised fetal movements and kick forces in a range of different intra-uterine conditions for the first time, quantifying the effects of these kicks at the local level of biomechanical stimulation at the hip joint. Crucially, this study provides a novel insight into the link between fetal biomechanics and hip joint morphogenesis, thus elucidating environments that predispose to hip joint malformations. Given the link between joint malformations and development of osteoarthritis, these findings could aid clinicians by informing the development of future preventative measures for neonatal joint conditions, such as DDH. This work has shed new light on a potential mechanobiological link between fetal kicks and the development of DDH, deepening our understanding of the aetiology of this condition and providing an explanation for changes in incidence under different intra-uterine conditions.

## Figures and Tables

**Supplementary Fig. 2:**